# Functions and Synthesis of Abscisic Acid (ABA) in Humans—Insights from Computational Approaches

**DOI:** 10.3390/ijms262211115

**Published:** 2025-11-17

**Authors:** Houda El-Maslahi, Ilona Turek, Chuyun Bi, Aloysius Wong, Oren Tzfadia, Helen Irving, Chris Gehring

**Affiliations:** 1 Department of Chemistry, Biochemistry and Biotechnology, University of Perugia, 06121 Perugia, Italy; houda.elmaslahi@outlook.com; 2 Australian Centre for Disease Preparedness, Commonwealth Scientific and Industrial Research Organisation, East Geelong, VIC 3220, Australia; ilona.turek@csiro.au; 3 Department of Biology, College of Science, Mathematics and Technology, Wenzhou-Kean University, Wenzhou 325060, China; bichuyun@wku.edu.cn (C.B.); alwong@kean.edu (A.W.); 4 Center for Integrative Plant Sciences, Wenzhou-Kean University, Wenzhou 325060, China; 5 Institute of Tropical Medicine, 2000 Antwerp, Belgium; 6 Holsworth Biomedical Research Center, Department of Rural Clinical Sciences, La Trobe University, Bendigo, VIC 3552, Australia; h.irving@latrobe.edu.au; 7 La Trobe Institute for Molecular Science, La Trobe University, Bendigo, VIC 3552, Australia

**Keywords:** abscisic acid (ABA), candidate ABA-binding proteins, ABA receptor, ABA synthesis, host defence, cancer, diabetes

## Abstract

Abscisic acid (ABA) is a “classical” plant hormone and is key to many plant responses, notably seed germination, transpiration and defence. It is becoming increasingly clear that ABA acts not just through the canonical PYL/PYR/RCAR receptors but also through other proteins that can interact specifically with ABA. Here we use genomic and transcriptomic resources to show that the human proteome also contains proteins with specific ABA-binding signatures and that some of these potential ABA-binding proteins may have roles in cancer and diabetes. In addition, there is evidence for the presence of ABA in humans; however, the source of it remains somewhat inconclusive. Here we propose an ABA synthesis pathway that, much like in fungi, does not include carotenoids but proceeds via farnesyl pyrophosphate. In summary, we review the current status of ABA research in *Homo sapiens* and propose avenues that might lead to novel insights into the synthesis and biological roles of this ancient hormone, e.g., in obesity and inflammation.

## 1. Introduction

The discovery of the chemical structure of the now considered classical hormone abscisic acid (ABA) [1] and the ABA-dependent physiological responses in higher plants were first reported more than 50 years ago, and ever since, the literature on ABA has steadily grown (>17,000 articles in PubMed (January 2025)). These articles detail aspects ranging from stimulus-induced ABA synthesis to ABA-dependent signalling and physiological responses at the molecular and systems level [2,3,4,5,6,7,8,9].

There is also increasing evidence for the presence and biological functions of ABA in animals including *Homo sapiens* notwithstanding the fact that ABA is a molecule originally associated with the regulation of cellular responses and biological processes in plants [7]. The universal presence of ABA has again been demonstrated by its detection in the nervous system of mice through a mass spectrometry assay [10]. However, it should be noted that the presence of ABA even in animals fed with an ABA-free diet fails to constitute proof that animals do produce ABA since the gut microbiome may contain enzymes capable of producing ABA [11].

The broad distribution of ABA among different groups of organisms suggests that its role as a signalling molecule emerged in the early stages of evolution [12]. It is hypothesized that ABA originated from a symbiotic organism, which would explain its ubiquitous presence across different taxa [11]. This distribution indicates that ABA may have played a crucial role in interspecific communication as early as the first multicellular life forms [13]. With evolution, these mechanisms were adapted and re-utilized, leading to the formation of complex regulatory systems in vastly different organisms, such as plants and animals [5]. ABA also modulates interactions between distinct organisms, such as in host–pathogen or mutualistic relationships [14]. This aspect opens new perspectives on its role as an ancient and universal signalling system capable of mediating interspecific communication with a significant impact on the survival and adaptation of organisms [13]. Recent research supports this hypothesis, indicating that ABA may act as a universal “stress hormone” performing similar functions in different organisms [15,16].

**Figure 1:**
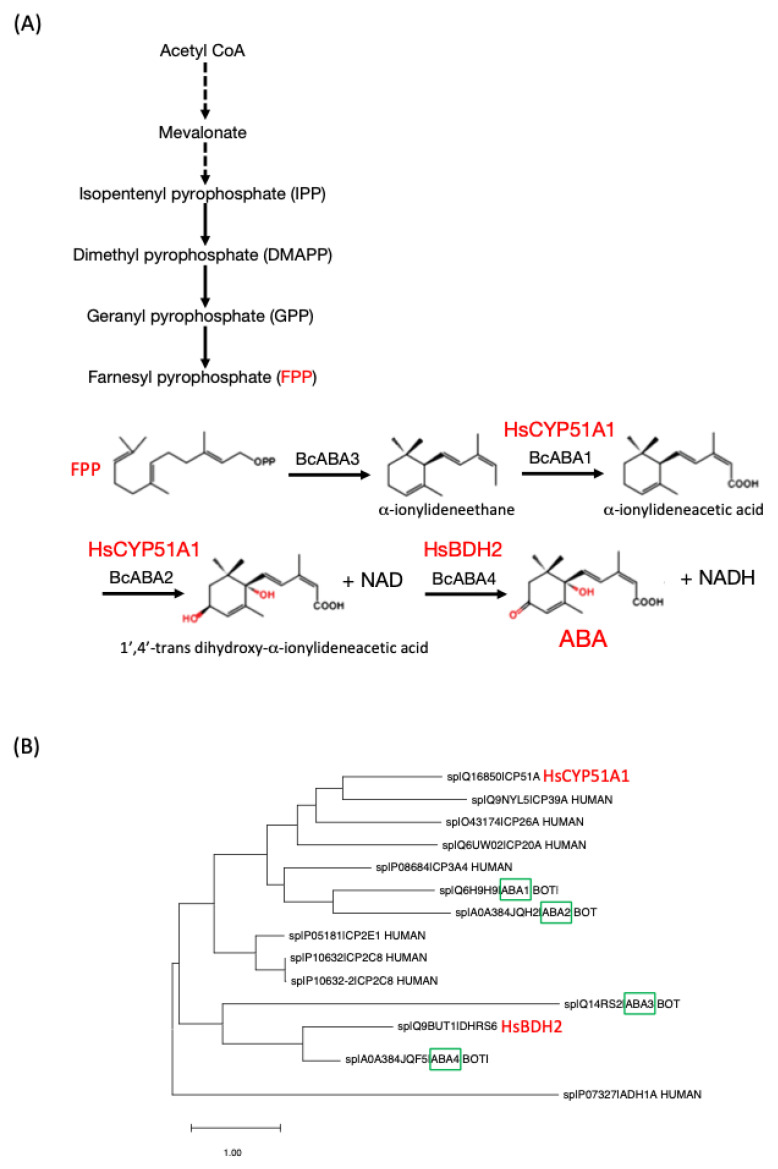
(**A**) Putative FPP-derived ABA pathway in *H. sapiens* reconstructed by orthology to the *Botrytis cinerea* (the anamorph form of *Botryotinia fuckeliana*) route. Fungal enzymes (black, lower) convert farnesyl-pyrophosphate to ABA via α-ionylideneethane and xanthoxin (BcABA1–4; green boxes). Directly above each fungal step, the closest human orthologue/close paralogue is shown in red supported by BLAST+ (version 2.17.0), domain conservation and phylogeny. (**B**) Phylogenetic relationship between the enzymes in (**A**) and an extended phylogenetic tree is shown in [5]. HsCYP51A1 and BcABA2 (19% identity, 34% positives and 18% gaps, with both proteins containing the PF00067 domain (Cytochrome P450) (2 × 10^−70^ and 5.1 × 10^−70^); BDH2 and BcABA4 (32% identity, 50% positives and 7% gaps, with both proteins containing the PF13561 domain (Enoyl-(acyl carrier protein) reductase) (1.9 × 10^−60^ and 5 × 10^−61^). (Additional human candidates (HsCYP2E1, HsCYP3A4, HsCYP26A1, HsADH1A/4) are also proposed based on co-expression/interaction data—see below).

In plants, ABA biosynthesis follows a complex and indirect pathway involving carotenoids [17]. The process begins with the synthesis of carotenoids and continues with their conversion into intermediate molecules, which are ultimately transformed into ABA through a series of enzymatic reactions [7,18,19]. In contrast, in fungi such as *Botrytis cinerea* (the anamorph form of *Botryotinia fuckeliana*), ABA biosynthesis occurs through a direct pathway based on sesquiterpenoids. Here, ABA is synthesized from farnesyl pyrophosphate (FPP; also referred to as “farnesyl-diphosphate”), a 15-carbon molecule produced via the mevalonate (MVA) pathway [20]. This mechanism differs significantly from the plant pathway, suggesting an independent evolution of ABA biosynthesis across different kingdoms of life [12] and notably in fungi (Figure 1).

Interest in ABA has markedly increased after research revealed its ability to affect the metabolism, cardiomyocytes and immune cells of animals (reviewed in [21,22]). ABA plays a key role in cellular signalling and the induction of apoptosis in cancer cells, sharing a signalling pathway involving nuclear receptors peroxisome-proliferator-activated receptor gamma (PPARGA) and retinoic acid receptor (RXRA) and does so by acting through its specific receptor LANCL2 [23]. ABA administration appears to offer many conceivable health benefits [16]. Clinical studies have shown that its administration may have positive effects in the treatment of type II diabetes [24,25,26], inflammatory intestinal diseases, depression [5] and neuroinflammation, suggesting its potential as a pharmacological agent [16]. These findings have driven further research to understand the mechanisms of ABA synthesis and signalling in mammals, with the goal of utilizing ABA as a possible therapeutic agent for metabolic and immunological diseases in humans [22,27,28].

Furthermore, recent studies have also raised questions about the origin of ABA in mammals [29]. Some studies suggest that ABA may be obtained through diet [27], while others hypothesize the existence of an endogenous synthesis mechanism [30]. Evidence supporting endogenous ABA synthesis has been strengthened by an experiment showing that plasma ABA levels increase after a glucose load in healthy human subjects [25,31], thus further demonstrating its involvement in the regulation of glucose metabolism in mammals [32].

Here we shall reconsider two unresolved questions. The first is, can ABA-binding (receptor-like) proteins be systematically detected in the human proteome? The second is, can humans synthesize ABA, and if so, what might the pathways be? A good start to answering the first question might be to adapt approaches that have proven promising in plant research [9]. In plants, the search began with the rather surprising finding that suggested that in *Vicia faba* guard cell membranes, an outward-rectifying potassium channel (GORK; At5g37500) was directly modulated by ABA and this in the absence of the canonical cytosolic ABA receptor [33]. This finding suggested that GORK may harbor an ABA-binding site through which this effect occurred. When we subsequently aligned GORK sequences from different species with the ABA-binding domain of the canonical PYL/PYR/RCAR receptor [34], we noted a significant degree of conservation in amino acids required for ABA receptor interactions. Subsequent experiments confirmed specific binding of ABA to the predicted site in GORK as well as ABA-induced K^+^-flux being dependent on the wildtype ABA-binding site of GORK. Furthermore, an amino acid search motif built based on conserved and functionally assigned residues in the ABA-binding domain [35,36,37] has enabled the identification of candidate ABA-binding proteins in the *Arabidopsis thaliana* proteome [9]. Several of these proteins are currently under experimental evaluation. It is noteworthy that the ABA-binding site is also present in the *A. thaliana* stelar K^+^ outward rectifier (AtSKOR) as well as GORK and SKOR channels in other dicots [33].

Given the apparent high conservation of ABA-binding sites and the fact that ABA causes various and significant changes in mammalian cells, we set out to identify candidate ABA-binding proteins in the human proteome. In addition, we used computational modelling [37,38] to evaluate these binding sites, and finally, we used functional meta-analyses of transcriptomic data to infer the possible roles of ABA in human physiology.

## 2. The Search for Candidate ABA-Binding Proteins in the Human Proteome

The search for candidate ABA-binding sites [9] in the human proteome with the stringent motif previously applied for the identification of *A. thaliana* ABA-binding proteins—D.{7,8}R.{3,4}D.{5,6}Y.{6,7}H—identified 20 proteins (Table 1). As always with computational predictions, further experimental programs are necessary to determine if these proteins do indeed bind ABA.

**Figure 2:**
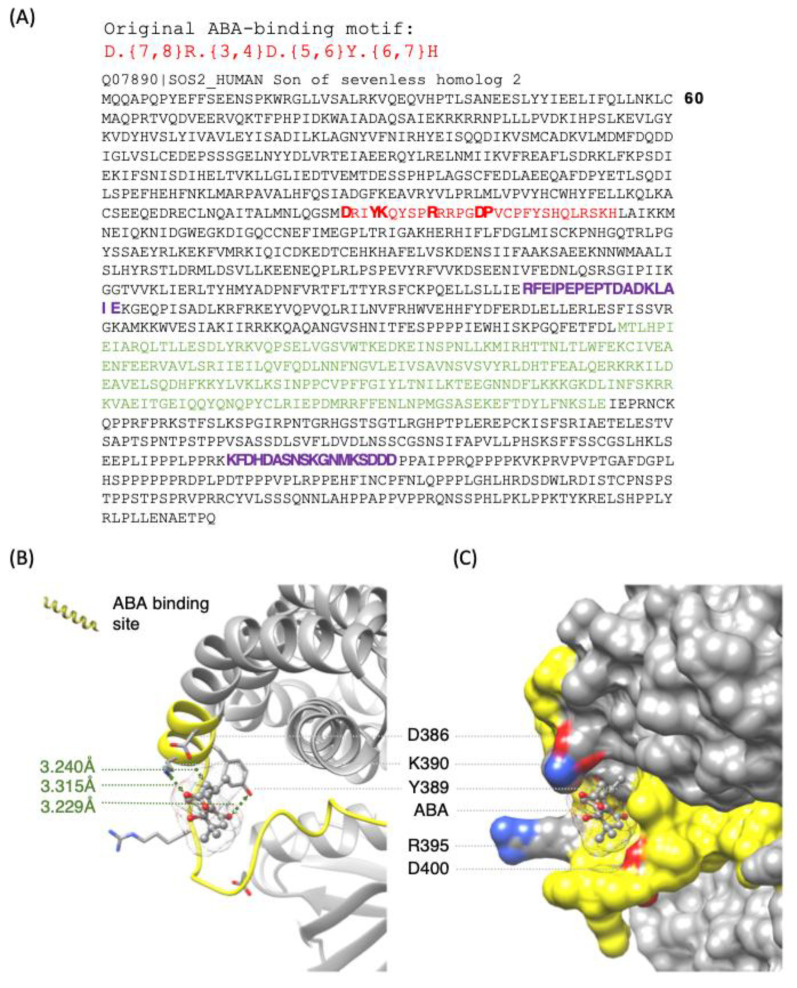
(**A**) ABA-binding motif and occurrence of the motif in SOS2 (marked in red). Marked in purple are two putative AC catalytic sites as defined by the term [RSK].[DE].{8,12}[RK].{1,3}[DE], and the green letters delineate a RasGEF domain. In the motif term, the letters in square brackets ([ ]) signify that any one of the amino acid in this position is allowed, full-stops (.) mean any amino acid is allowed, and curly brackets ({}) delineate the range (number of amino acids, e.g., {1,3} stands for at least one amino acid and not more than 3). (**B**,**C**) Ribbon (left) and surface (right) models of SOS2 docked with ABA. Intermolecular distances between key amino acids of the ABA motif (yellow) and ABA are represented in green, while key amino acids in the motif are coloured by elements and labelled accordingly. The SOS2 model was obtained from AlphaFold [40] which is available at https://alphafold.ebi.ac.uk/entry/Q07890 (accessed on 01/03/2024). Docking simulations were conducted using AutoDock Vina (version 1.1.2) [41], and structural visualization and image preparation were performed using UCSF Chimera [42].

It is noteworthy that a slightly relaxed search motif—D.{7,8}R.{8,10}Y.{6,7}H—is also present in LANCL2, which has previously been identified as an ABA receptor [30,39]. This would indeed suggest that our ABA-binding candidate list (Table 1) may be far from exhaustive. Four of the ABA-binding candidates are annotated as DNAJ proteins, which operate as molecular co-chaperones that recruit DnaK family chaperones (heat shock family) to enable protein folding, protein transport or the remodelling of protein complexes. Two ABA-binding candidates (FCGR2C and SOS homolog 2 (SOS2)) are associated with B-cell receptor signalling. Here we use SOS2 as an example for further structural and computational evaluations [40,41,42] to investigate the feasibility of accommodating ABA at the predicted site [35,36]. The region encompassing the ABA motif in SOS2 assumes a pocket that can spatially accommodate ABA. Molecular docking analysis showed that ABA could dock at this pocket with a free energy of −5.6 kcal/mol and has a binding pose that is oriented towards key amino acids in the motif (D386, Y389 and K390) with intermolecular distances measured at 3.240, 3.229 and 3.315 Å from the ligand, respectively, thus lending some credence to SOS2 possibly interacting with ABA (Figure 2). While −5.6 kcal/mol is not a strong affinity, this may be advantageous since the ligand may more easily move off the protein as concentrations decrease and consequently cease allosterically modulating the activity.

SOS2 also harbours two adenylate cyclase (AC) catalytic centres [43,44,45,46,47] as defined by the conserved amino acid motif present in many annotated and/or experimentally tested ACs: [RSK][YFW][DE].{8,12}[RK].{1,3}[DE]. The SOS2 also contains a RasGEF domain [48] located between the AC catalytic centres, while the SOS2 and vinexin contain annotated SRC homology 3 (SH3) domains [49,50]. SH3 domains are thought to enable protein–protein interactions in the signal transduction pathway [51], thereby modulating signal output. Given that ABA triggers and/or modulates many signalling pathways in plants [7,9] and also humans [22], we queried if more ABA-binding candidates contained hitherto unreported SH3 domains. To this end we assembled a SH3 consensus amino acid motif—[VILAR]P.[VILAR]P—consisting of one or two central prolines with aliphatic amino acids left and right and flanked by another C-terminal proline. We found that such a motif was present in nine of the ABA-binding candidate proteins (Table 1) but none of the DNAJ proteins.

Another interesting ABA-binding candidate protein is the salvador homolog 1 (SAV1), which is a regulator of the kinases STK3/MST2 and STK4/MST1 in the Hippo signalling pathway. This pathway functions in organ-size control and tumour suppression and does so by restricting proliferation and promoting apoptosis [52].

Naturally these ABA-binding candidates are based on predictions, and future experimental programs are required to verify the predictions and assess the biological relevance of ABA binding in vivo. We foresee that the selected candidate proteins will first be cloned and expressed for in vitro ABA-binding studies as detailed previously [33]. To establish binding specificity, mutants with modified ABA binding sites (motifs) will also be tested to see if reduced affinities result. As an additional control, reduced binding should also occur with the biologically inactive (-)ABA isomer [33]. A next step will involve in vitro studies of biochemical effects on the binding protein. If the binding protein is, for example, an enzyme, it will be telling to test possible modifying effect(s) of ABA on this enzyme activity. Once ABA-binding and the functional relevance of such binding have been elucidated, targeted metabolomics of, for example, human hepatocyte and/or β-cell cultures (in the presence or absence of added ABA), targeting substrates and products of ABA-modified enzymes might provide valuable insights into the role of ABA at the systems level.

Furthermore, experimentally confirmed ABA-binding candidates with obvious links to clinically relevant syndromes such as diabetes, cancer or immune responses might help us to discover novel drug targets and may open the way for testing some ABA analogues or agonists [26,53] with potentially more efficient binding to the proposed sites.

## 3. Computational Approaches to the Elucidation of ABA Synthesis in Humans

If we assume that animals including *H. sapiens* can make ABA while keeping in mind that the synthesis pathway via carotenoids is unlikely [22,54], then a reasonable hypothesis is that ABA is synthesised via a pathway similar to that evolved in fungi (Figure 1). In the necrotrophic fungus *Botrytis cinerea* (the anamorph form of *Botryotinia fuckeliana*) the synthesis proceeds from FPP, which is present in humans [55]. The candidate ABA synthesising enzymes (see Figure 1) include BcABA1 (Cytochrome P450 monooxygenase aba1, UniProt identifier: Q6H9H9), BcABA2 (Cytochrome P450 monooxygenase aba2; UniProt identifier: A0A384JQH2), BcABA3 (α-ionylideneethane synthase aba3; UniProt identifier: Q14RS2), and BcABA4 (Short-chain dehydrogenase/reductase aba4; UniProt identifier: A0A384JQF5) (see Figure 1). BcABA4 in fungi belongs to the same phylogenetic branch as the human HsBDH2 (Binding Dehydrogenase 2, UniProt identifier: Q9BUT1), while BcABA2 is located on the same branch as HsCYP51A1 (Lanosterol 14-α demethylase; UniProt identifier: Q16850). This phylogenetic branch is also close to BcABA1, suggesting a significant analogy in ABA biosynthetic pathways between fungi and animals [5].

In a first step to discover ABA synthesis candidates, we mapped the strongest phylogeny-anchored orthologues (HsCYP51A1 → BcABA1/BcABA2; HsBDH2 → BcABA4) (see Figure 1). In a second step, we applied co-expression/association evidence to establish a broader set of enzymes (CYP2E1, CYP3A4, CYP26A1, ADH1A/4) as possible candidates that have a role in the tissue-specific routing of FPP-derived intermediates with possible roles in ABA synthesis.

To further investigate the candidate orthologues, we used a three-step protocol with a view to put these candidates in a functional context which might afford further insights into the pathways in which they operate.

First, genes that execute the same biochemical pathway are typically co-regulated across a large compendia of samples, and this manifests as correlated mRNA abundance (“guilt by association”). Such correlations are not anecdotal: co-expressed gene sets are systematically enriched for shared functions and often recover known protein complexes and enzymatic chains [56,57].

Second, when co-expression is estimated from many independent datasets and summarized as a network, stable modules emerge that capture pathway structure with useful accuracy. Large-scale evaluations show that stringent edge filtering markedly improves functional coherence and reproducibility of the inferred nodules [58].

Third, STRING’s “co-expression” channel operationalizes this principle at scale by aggregating multiple transcriptome resources and then integrating them with orthogonal evidence (experiments, curated databases and literature) into a calibrated combined score. Using a high combined-score threshold therefore prioritizes associations that recur across datasets and evidence types, making co-expression a robust prioritization device for pathway membership rather than a standalone proof of mechanism. In our context, if human orthologues/close paralogues of the fungal ABA enzymes showed tight co-expression with each other and with enzymes from the mevalonate/retinoid subnetworks, this would provide orthogonal, data-driven support for their joint participation in an endogenous, FPP-derived ABA route.

**Figure 3:**
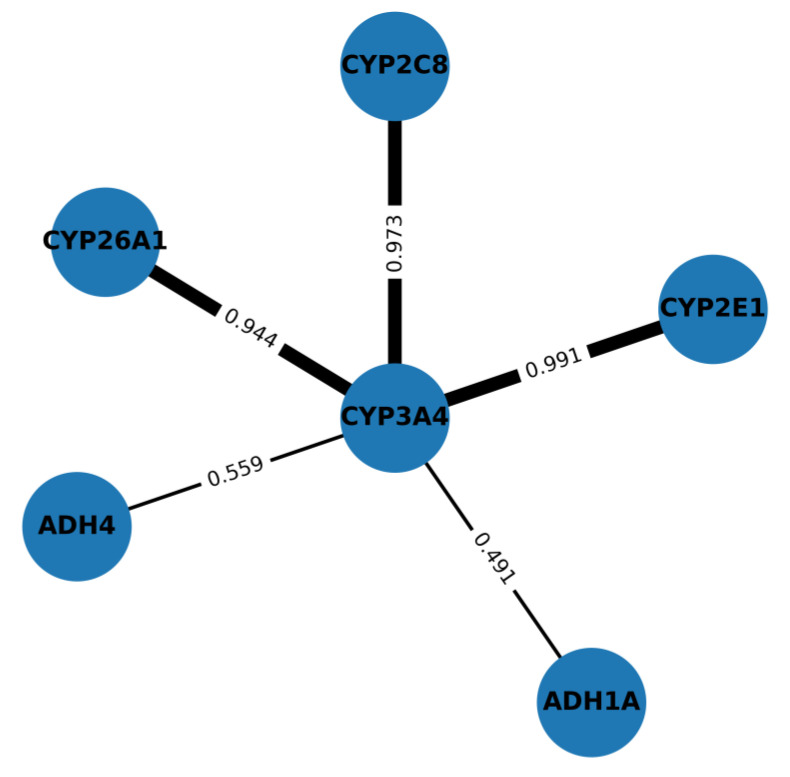
STRING analyses of the protein interaction network of human candidate genes involved in ABA synthesis. Nodes = proteins; edges = STRING combined scores. Edge width: thick ≥ 0.900; medium = 0.700–0.899. Only edges with co-expression ≥ 0.400 are displayed. Human CYP3A4 is a central hub with high-confidence associations to CYP2E1 (0.991), CYP2C8 (0.973) and CYP26A1 (0.944) and moderate links to ADH4 (0.559) and ADH1A (0.491). The methods used are detailed in the text.

Specifically, based on co-expression [59], functional and physical interactions, we identified HsCYP2E1 (UniProt identifier: P05181), HsCYP26A1 (UniProt identifier: O43174), HsCYP3A4 (UniProt identifier: P08684), HsADH1A (UniProt identifier: P07327), HsADH4 (UniProt identifier: P08319) and HsCYP2C8 as the interaction partners associated with ABA biosynthesis (Figure 3). To generate the interaction network, we used STRING v12.0 for *Homo sapiens* queries. Unless stated otherwise, evidence channels included co-expression, experiments, curated databases, neighbourhood, gene fusion, co-occurrence and text-mining. The minimum combined score was set to 0.700 (high confidence), with Markov Clustering (MCL) inflation = 3. For co-expression-focused views, edges with co-expression score ≥ 0.400 were retained. Node/edge attributes (combined and channel-specific scores) were exported to Cytoscape v3.10.0; edge width scales were combined with the score. Functional enrichment (GO-BP, KEGG) used STRING’s FDR-corrected tests, with FDR < 0.05 being retained and network statistics (average node degree, clustering coefficient, and PPI enrichment *p*-value) for the protein queries being reported.

The neighbouring proteins of ADH4, such as HsCYP2E1 and HsCYP26A1, are associated with the metabolism of xenobiotics through the cytochrome P450 system, a pathway annotated in the KEGG database as “Drug Metabolism”. Other related proteins, including HsCYP3A4, HsADH1A, HsADH4 and HsCYP2C8 (UniProt identifier: P10632), are frequently co-expressed or mentioned together in other organisms, consistent with evolutionary and functional conservation. For the identified genes, co-expression analyses were conducted using the STRING platform (Ref. [59] and references therein), with a selective filter for biological pathways potentially associated with ABA synthesis being applied. Specifically, the pathways of terpenoids, isoprenoids and retinoic acid were examined.

## 4. Identification and Characterisation of Transcription Factors Regulating the Identified ABA Synthesis Genes

The analysis of transcription factors revealed their significant role in regulating genes associated with key metabolic processes, particularly terpenoid biosynthesis and secondary (drug) metabolism. Perhaps unsurprisingly, PPARGA (Peroxisome Proliferator-Activated Receptor Gamma) and RXRA (Retinoid X Receptor α; UniProt identifier: P19793) were identified as they have been previously implicated in ABA signalling. ABA binds and activates PPARGA [60,61] and inhibits RXRA signalling [23]. Both PPARGA and RXRA interact to activate HMGCS1 (3-hydroxy-3-methylglutaryl-CoA synthase 1; UniProt identifier: Q01581), a critical enzyme in the mevalonate (MVA) pathway, which governs terpenoid biosynthesis (KEGG pathway map03320) and modulates β-oxidation.

Furthermore, Peroxisome Proliferator-Activated Receptor (PPAR) signalling (in cooperation with NR1I2 (Pregnane X Receptor, PXR; UniProt identifier: O75469) and RXRA was identified as modulator of *HsCYP3A4* expression. It is noteworthy that HsCYP3A4 is a member of the HsCYP51A1 family, which has a role in hepatic drug metabolism. This again is consistent with previous findings that implicate NR1I2-RXRA heterodimers in the regulation of drug-detoxifying enzymes, thereby influencing xenobiotic metabolism and pharmacokinetics [62]. Taken together, these findings are consistent with a potential link between these transcriptional regulators and ABA synthesis and ABA-dependent stress signalling.

## 5. ABA-Binding Candidates as Key to Inferring Novel ABA Functions in Humans

ABA has been implicated as a factor in several human disorders including neurodegenerative diseases, cancer, type 2 diabetes and immune dysfunctions (for reviews see [21,22]). From this perspective, we identified 20 novel candidate ABA-binding proteins whose function in ABA signalling has not been previously considered, and below, we speculate about how ABA may contribute to their function. A quarter of the proteins identified as ABA-binding proteins here are annotated as DNAJ (also referred to as “J-proteins” or “HSP40s”) homologs; three of these four DNAJ proteins belong to DNAJB (type II with a total of 13 members in the human proteome), and one belongs to DNAJC (type III with a total of 32 members in the human) family. DNAJ proteins are defined by the presence of a highly conserved ~70-amino-acid-long J domain containing four α-helices, with a tertiary structure that resembles a “protruding finger” (helix 2 and 3). Further, a highly conserved histidine–proline–aspartic acid (HSP) motif is located on the loop between helix 2 and 3 [63]. Both helix 2 and the HSP motif are important for the interaction with partner HSP70s, and the motif is required for stimulation of the HSP70 ATPase activity [64]. Interestingly, the ABA-binding site identified in each of these DNAJ proteins occurs in helices 1 and 2, with the last amino acid residue of the ABA-binding site (H) being part of the HSP motif. Such close proximity of the ABA-binding site to the HSP motif suggests the possibility that interactions of ABA with the DNAJ proteins can modulate molecular processes ranging from the stimulation of the ATPase activity of HSP70 to localized regulation of the HSP70 polypeptide binding and release cycle [65] and to potential chaperoning activities of DNAJ proteins that are independent of HSP70 [66]. If so, this could impact the DNAJ support of the chaperoning network to maintain proteostasis and consequently the disease pathologies associated with defects in protein homeostasis. For instance, DNAJB1-HSP70 prevents NO-mediated apoptosis in macrophages through binding to and inhibiting translocation of the BCL2 associated X apoptosis regulator (Bax) to mitochondria [67,68]. Multiple studies have implicated ABA in ameliorating inflammatory immune responses partly through PPARG effects [24,60], and this may be via modifying the DNAJB1-HSP70 complex. These responses could be important in combating viral infections in which DNAJB1 has been implicated (e.g., suppressing hepatitis B virus replication [69] while promoting replication of influenza A virus [70], human immunodeficiency virus [71] and human cytomegalovirus [72]).

Other pathologies associated with protein homeostasis defects include neurodegenerative diseases and type 2 diabetes and cancer, to all of which DNAJs contribute [63,73,74]. Notably, DNAJ(B1) activates ABA binding to HSP70 proteins, and ABA can specifically increase ATPase activity [75]. The function of DNAJBs, and DNAJB1 in particular, in modulating protein aggregation and especially the amyloid fibrils in a number of neurodegenerative diseases has been extensively studied [76]. Complete suppression of Huntington (HTT) fibrillization can be achieved by DNAJB1, HSC70 and HSP110 complex [77,78]. Meanwhile, in Parkinson’s disease, DNAJB1 is co-localized with α-synuclein (αSyn) inclusions in cells [79,80]. DNAJB1 has been implicated in Alzheimer’s disease, where it suppresses tau aggregation and alters morphology of tau amyloids [81,82]. Interestingly, pre-clinical animal model studies indicated that ABA administration improves symptoms of neurological diseases including Alzheimer’s disease and Parkinson’s disease [83,84,85,86,87]. Although LANCL2 signalling has been implicated at least in regulating neuropathic pain [85,88], it is therefore enticing to speculate that ABA binding sites in DNAJB proteins may also form a contributing mechanism.

DNAJB1 has been implicated in multiple cancers including triple-negative breast cancer [89], cholangiocarcinoma [90] and pancreatic cancer [91], to name just a few. DNAJB1 is also implicated in castration-resistant prostate cancer (CRPC), characterised by reactivation of androgen receptor signalling and resistance to standard antiandrogen therapy. This may make the DNAJB1/HSP70 axis a novel CRPC treatment target [92], and it is of interest to investigate how ABA might affect this axis. Various cancers have been associated with DNAJB12 [91,93] and DNAJC9 [91,92,94], which also contain predicted ABA-binding sites, but the roles of these sites require investigation. Meanwhile, DNAJB13 is necessary for the formation and function of the ciliary and flagellar axonemes required for male infertility and normal airway function. Notably, a splicing defect caused by a stop codon substitution of tyrosine 24 (Tyr24) in the J domain (and the predicted ABA-binding site) of DNAJB13 results in a typical clinical picture of primary ciliary dyskinesia, associated with chronic airway infections [95].

SAV1 mainly acts as a scaffold in the tumour-suppressor Hippo pathway that regulates cell proliferation and cell death and is one of the key signal pathways that controls carcinogenesis. SAV1 promotes Hippo activation [52,96] and is an interactor of mammalian sterile-20-like kinase 1 (MST1) that augments MST1-induced apoptosis [97]. Therefore, it is not surprising that SAV1 has tumour-suppressive roles and that its low expression in cancer is associated with poor prognosis [94]. The human SAV1 (see Table 1) contains an N-terminal flexible domain, two tandem WW domains (aka rsp5-domain) mediating dimerization and a C-terminal SARAH (Sav/Rassf/Hpo) domain mediating heterodimerization with STK3 (aka MST2). Interestingly, the ABA-binding site identified in our search in the human SAV1 sequence (aa 137–163) is localised in the N-terminal flexible domain of the protein. This N-terminal domain (aa 1–199) is required and sufficient for the direct binding with the intracellular domain of the cell adhesion molecule KIRREL1 (aka NEPH1) that leads to recruitment of SAV1 at cell–cell contact sites, thereby enhancing the activation of the Hippo pathway [98]. Furthermore, the predicted ABA-binding site is adjacent to the hydrophobic-filamin-A (FLNA)-binding motif (aa 116–124), and its disruption alters subcellular localization of SAV1 [99]. It is conceivable that ABA binding to the N-terminal flexible domain of SAV1 could impact the mechanism by which the Hippo pathway senses cell–cell interactions at the plasma membrane.

**Figure 4:**
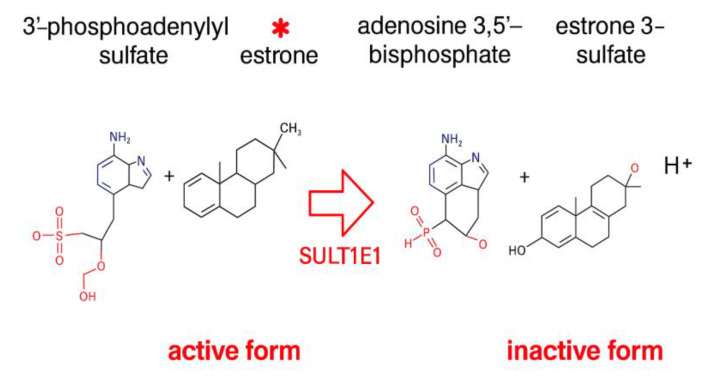
Catalytic reaction of the ABA-binding candidate sulfotransferase 1E1 (SULT1E1) that catalyses the reaction of the active estrone E1 (red asterisk) to the inactive estrone 3-sulfate.

Regarding the link between cancer and ABA, it is worthwhile to single out the sulfotransferase 1E1 (SULT1E1) (see Table 1). This enzyme is also an ABA-binding candidate (see Table 1) and catalyses a reaction that converts estrone to estrone 3-sulfate (Figure 4). Estrone (E1) (sometimes spelt “oestrone”) is a weak estrogen hormone produced primarily by adipose tissue after menopause. This steroid hormone promotes sexual development and the development of the reproductive system and is also used in hormone replacement therapy. Estrone is present in both sexes and, and in women becomes the dominant estrogen after menopause [100,101].

It has recently been demonstrated that the major premenopausal estrogen, 17β-estradiol, and postmenopausal estrone can have opposing roles to inhibit or indeed drive the tumour-promoting effects of inflammation and obesity [102]. Estrone (Figure 4) was reported to be proinflammatory as well as pro-oncogenic. Estrone also links metabolism and cancer in that it increases with obesity and stimulates expansion of stem-like cells in hormone-sensitive breast cancer, which promotes tumour growth [102]. It is therefore conceivable that SULT1E1 converting the biologically active estrone into the inactive estrone 3-sulfate can modify and possibly attenuate the rate of cell proliferation in hormone-sensitive breast cancers, particularly so if ABA binding to SULT1E1 enhances catalytic efficiency.

Finally, it should be noted that several online tools in the public domain allow for the inspection of expression data in vertebrates both at the level of the transcriptome and the proteome. Importantly, the databases allow extraction of expression profiles for genes and proteins under pathological conditions such as tumours, e.g., [103,104]. Protein searches can easily be performed online (https://www.proteinatlas.org; accessed on 01/03/2024) and yield information on the tissue-specific and, for example, tumour-dependent expression of proteins. Such information is useful in the selection of ABA-binding candidates before experimental studies are initiated.

Taken together, the findings indicate that these ABA-binding candidates make promising targets for future in vitro and in vivo studies that may offer new insights into the mechanism of action and possible therapeutic applications of an ancient stress response system.

## 6. ABA Synthesis in *H. sapiens*—An Outlook

Although vertebrates lack the carotenoid cleavage route central to ABA formation in higher plants [105], our multi-layered screen converges on a credible alternative pathway that mirrors the sesquiterpenoid route employed by *B. cinerea* [20,106,107]. Starting from the endogenous metabolite farnesyl-pyrophosphate (FPP), we identified a minimal four-step reaction sequence catalysed by human orthologues (or close paralogues) of BcABA1–4. The strongest candidates—CYP51A1, CYP2E1, BDH2, ADH1A/4 and CYP26A1/3A4—are (i) phylogenetically proximate to the fungal enzymes, (ii) co-expressed in tissues that accumulate ABA (in the pancreas, liver and brain) and (iii) are embedded in the mevalonate/retinoid subnetworks that supply FPP and retinoic acid. Taken together, these observations lend plausibility to an endogenous vertebrate ABA biosynthetic route that is FPP-derived rather than carotenoid-derived. The most direct way to test the presence of such an FPP-derived pathway will require the synthesis of recombinant human candidate enzymes, e.g., HsCYP51A1 (see Figure 1), and testing them for enzymatic activity in vitro.

Transcription factor enrichment further strengthens such a hypothesis. PPARγ/RXRα and NR1I2 (PXR) can conceivably operate as regulators of lipid, isoprenoid and xenobiotic metabolism to thereby co-activate *CYP3A4*, *CYP2E1* and *HMGCS1* and functionally link ABA-synthesis enzymes with the FPP-generating mevalonate pathway and retinoid signalling. This regulatory integration is striking because PPARγ and RXRα have both been implicated in ABA binding and ABA-responsive glycaemic control and anti-inflammatory actions [22,23,60,61]. Hence, ABA biosynthesis, retinoid turnover and PPAR/PXR signalling appear to form an interconnected metabolic circuit that could explain the rapid rise in circulating ABA after glucose load.

Furthermore, the phylogenetic, co-expression and regulatory data converge on a testable working model: mammals operate a latent, fungal-like ABA synthesis pathway that depends on the abundant FPP pool and is inducible under metabolic or xenobiotic stress. Experimental falsification should now focus on the following: (a) targeted metabolomics of presumed intermediates of the proposed ABA synthesis pathway (α-ionylideneethane, α-ionylideneacetic acid and/or 1′,4′-trans dihydroxy-α-ionylideneacetic acid) in, for example, human hepatocyte and/or β-cell cultures; (b) CRISPR ablation or pharmacological inhibition of the top candidate enzymes (Figure 1), followed by ABA quantification; and (c) isotope-tracer studies (^13^C-mevalonate → ABA) in vivo. In addition, cell lines with deletions in one or several of the proposed synthesis genes (Figure 1) will enable biochemical and cell biological validation of our hypothesized pathway. Such validation would establish the first critical experimental link between endogenous ABA and human physiology. This in turn may open a door for rational ABA-based therapeutics to the treatment of some forms of diabetes, inflammation and some cancers.

## Figures and Tables

**Table 1:** Candidate ABA-binding proteins identified in the human proteome.

UniProt ID	Name of the Protein
O60504	Vinexin (SH3 domain cont. annotated)
O60583	Cyclin-T2
O94875 ^&^	Sorbin (SH3 domain cont. annotated)
P25685 *	DNAJ homolog subfamily B member 1 (DNAJB1)
P31995 **	Low aff. IG γ Fc region receptor II-c (FCGR2C)
P49888	Sulfotransferase 1E1 (SULT1E1)
P59910 *	DNAJ homolog subfamily B member 13 (DNAJB13)
Q07890 **^,#,&^	Son of sevenless (SOS) homolog 2 (SOS2)
Q14C86	GTPase-activating protein & VPS9 domain
Q16394 ^&^	Exostosin-1
Q7RTN6	STE20-related kinase adapter protein
Q8N5S9 ^&^	CaM-dependent protein kinase 1
Q8WXX5 *	DNAJ homolog subfamily C member 9 (DNAJC9)
Q92839	Hyaluronan synthase
Q9H4B6 ^&^	Protein salvador homolog 1 (SAV1, hWW45)
Q9HAC8 ^&^	Ubiquitin domain-containing protein
Q9NXW2 *	DNAJ homolog subfamily B member 12 (DNAJB12)
Q9NYQ8 ^&^	Protocadherin Fat 2
Q9UKJ3 ^&^	G patch domain-containing protein 8
Q9Y2K2 ^&^	Serine/threonine-protein kinase SIK3

* GO categories: DNAJ is 96.9-fold enriched (FDR 1.30 × 10^−3^); ** Associated with B-cell receptor signalling; ^#^ The protein harbours an adenylate cyclase catalytic centre; ^&^ The SH3 domain motif ([VILAR]P.[VILAR]P) is present.

## Data Availability

No new data were created or analyzed in this study. Data sharing is not applicable to this article.

## Abbreviations

The following abbreviations are used in this manuscript:

ABA	abscisic acid
FPP	farnesyl pyrophosphate
PPARGA	peroxisome-proliferator-activated receptor gamma
RAXRA	retinoic acid receptor
CYP	cytochrome P450
GORK	outward-rectifying potassium channel
